# Registered nurses' perceptions of having a calling to nursing: A mixed‐method study

**DOI:** 10.1111/jan.15157

**Published:** 2022-02-21

**Authors:** Hanna Kallio, Mari Kangasniemi, Marja Hult

**Affiliations:** ^1^ Department of Nursing Science, Faculty of Medicine University of Turku Turku Finland; ^2^ Department of Nursing Science, Faculty of Health Sciences University of Eastern Finland Kuopio Finland

**Keywords:** calling, care, mixed methods, nursing, registered nurses, working life

## Abstract

**Aim:**

To explore how nurses perceived having a calling to nursing.

**Design:**

A mixed‐method study.

**Methods:**

Survey data collected in autumn 2020 and semi‐structured individual interview data collected in spring 2021. The 7925 survey respondents were care professionals and 414 of them were registered nurses. The 23 interview participants were registered nurses who responded to the survey. We examined the survey results using analysis of variance and *t*‐tests and the interview data with qualitative thematic analysis.

**Results:**

Registered nurses had a lower calling than other care professionals. Based on the interviews, having a calling to nursing produce four key findings. Nurses with a calling experienced their work as meaningful. They also adopted a humane and holistic approach to their work. However, their calling could change during their professional career. Due to its historical roots, having a calling was seen as a risk for the nursing profession, as it meant nurses had an oppressed position in society and nurses associated it with poor working conditions and low pay.

**Conclusion:**

Our study showed that having a calling to nursing had multidimensional benefits for the individual nurse, their patient, colleagues, organization and society, but showed strong association between calling and nurses' poor working conditions and low pay.

**Impact:**

We found that nurses had a lower calling than other care professionals. Calling still exists, but it can produce tension in modern nursing. Organizations and society need to focus on how calling can be seen as a more positive attribute of nursing and improve nurses' working conditions and pay.

## 1 INTRODUCTION

Nurses' work engagement has become increasingly topical in recent years. Discussions have focused on their working conditions (McDermid et al., 2020) and, in particular, their workload (McHugh & Ma, 2014; Racy et al., 2021). Care has increased due to the rapid ageing of the general public (United Nations, 2019) and the increasing health hazards caused by climate change (Catton, 2020). At the same time, the number of nurses has decreased (Drennan & Ross, 2019) due to retention issues and the fact that the profession has become less attractive (Xie et al., 2021). Nurses' workloads have got heavier and their dissatisfaction with their working life has increased (Ahlstedt et al., 2019; McDermid et al., 2020). Traditionally, nursing has been described as ‘a calling profession’, referring to passionate desire to become nurses and serve others (Emerson, 2017). Very little has been known about calling in contemporary nursing. It was important to establish this because of the changes that have taken place in nursing, including the workforce issues.

## 2 BACKGROUND

The concept of calling relates to the meaning that people attach to work goals. These can be defined as classical (Ponton et al., 2014), modern (Dobrow & Tosti‐Kharas, 2011) and neoclassical (Dik & Duffy, 2009) orientations. The classical definition of calling refers to a professional's destiny and duty and it has traditionally been based on a person's desire to fulfil God's will in life. The modern definition of calling relates to choosing a career that provides fulfilment in life. (Ponton et al., 2014.) It has been described as a consuming, meaningful personal experience (Dobrow & Tosti‐Kharas, 2011). From a neoclassical perspective, calling (Ponton et al., 2014) has been understood as a three‐dimensional construct. The first dimension is a transcendent summons, which is an external force that can originate, for example, from either God or the needs of society. The second is purposeful work, which includes the purpose and meaningfulness of work and may provide life with meaning. The third dimension is prosocial orientation, which refers back to the classical definition, by stating that meaningfulness is derived from serving the common good or the well‐being of society (Dik & Duffy, 2009).

Based on previous research, calling has been a common reason for people entering the nursing profession (Eley et al., 2012) and staying in it (Xu et al., 2020). Being called to nursing has been defined as a person's intrinsic aspiration to engage in nursing practice, which centres around meaningful work (Emerson, 2017). Having a calling has been found to boost nurses' work motivation (Ziedelis, 2019), job satisfaction (Xu et al., 2020) and work engagement (Afsar et al., 2018) and empower them to cope with work demands (Ziedelis, 2019). It has also been shown to advance the sense of coherence and study success (Colomer‐Pérez et al., 2019). Calling has often been connected to compassion (Carter, 2014; Emerson, 2017) and to patients' experiences of good care (White, 2002) and care quality (Zhou et al., 2021).

Studies on how having a calling affects a nurse's ability to cope have been contradictory. People who have a calling are more likely to become workaholics (Choi et al., 2020). They are also more vulnerable to the growing risk of fatigue, as they might sacrifice their time and well‐being for work (Bunderson & Thompson, 2009). Previous research has also recognized certain conflicts in how nurses have perceived their calling in relation to employment, as some of them have described nursing as a financial necessity rather than ‘a luxury that they could afford’ (Carter, 2014, 702). Nurses' calling has been presented in media as justification for their abuse (e.g. Kenny, 2018; Niemonen, 2020), but the current scientific literature does not describe how nurses themselves perceive their calling in relation to contemporary nursing. The need to approach calling in a wide and unprejudiced manner has been brought up (Carter, 2014) when exploring nurses' motives for their work (Ahlstedt et al., 2019).

## 3 THE STUDY

### 3.1 Aim

The aim of the study was to explore how nurses perceived being called to nursing. The research questions focused on the current state of nurses' calling and what it meant to their work.

### 3.2 Design

This was a mixed‐method study that used segregated design with sequential synthesis using two‐phase approach (Noyes et al., 2019). In the first phase, the quantitative component provided knowledge of the current state of nurses' calling. This part included nurses and other care professionals and revealed nurses' lower calling. Thus, its result informed the second phase (Noyes et al., 2019), a qualitative investigation, that was needed to provide a deeper insight on what calling meant to nurses' work. Analysis methods were separate so that the quantitative analysis first answered the sub‐question about the current state of nurses' calling and then the qualitative analysis answered to what calling meant to their work. Integration of the results occurred in the discussion (Noyes et al., 2019) in which we scrutinized quantitative and qualitative findings in relation to each other.

### 3.3 Research environment

In Finland, registered nurses spend 3.5 years studying for a Bachelor's degree at a university of applied sciences. The number of applicants for nursing education has decreased steadily in recent years. In 2018, there were 61,772 applicants in Finland (Statistics Finland, 2021) and 92% were women. Approximately one‐third (32%) of Finnish nurses are under the age of 36, half (50%) are aged 36–55 and the rest are older. The majority (80%) of nurses work in healthcare and 17% work in social care. Finnish nurses receive lower salaries than their Nordic colleagues (Nurses' Association, 2021). There are shortages of nurses all over Finland (Occupational Barometer, 2021). About 90% of nurses belong to one of the trade unions in Finland (Findicator, 2020).

### 3.4 Participants and recruitment

The survey used systematic total sampling (Grove et al., 2013) of the 93,000 members of two Finnish trade unions for care workers and one company that provided temporary staff in the care sector. The researcher liaised with the key contacts in the three organizations, who emailed an invitation letter to their members directly or as part of their monthly newsletter. Of the 7925 (9%) care professionals who enrolled in the study, 414 were registered nurses and the rest were other care professionals (Table 1). At the end of the survey, we asked if the respondents would be prepared to take part in interviews and 403 agreed. We used systematic selection (Grove et al., 2013) and invited all 34 registered nurses who were members of one of the trade unions. Of these, 20 agreed to take part in the interviews and three pilot interviews were also included.

**TABLE 1:** Means (*SD*) for dimensions of calling by the groups of care professional

	Transcendent summons		Purposeful work		Prosocial orientation		CVQ total	
	*M*	*SD*	*M*	*SD*	*M*	*SD*	*M*	*SD*
Registered nurses (*n* = 414)	9.03*	2.60	9.74*	2.99	11.81*	2.57	10.19*	2.30
Practical nurses (*n* = 5453)	9.70	2.34	9.90	3.06	11.91	2.64	10.50	2.29
Social advisors and assistants (*n* = 1506)	9.93	2.27	10.17	3.02	12.40	2.47	10.83	2.19
Early education teachers (*n* = 140)	9.86	2.25	10.39	3.03	12.42	2.28	10.89	2.12
Managers (*n* = 78)	9.85	2.53	10.65	2.86	12.59	2,66	11.03	2.22
Others^a^ (*n* = 189)	9.34	2.53	10.02	3.41	11.90	3.00	10.42	2.54

Abbreviation: CVQ, Calling and Vocation Questionnaire.

^a^ Secretaries, cleaners, instrument attendants, etc.

* *p* ≤ .01, when tested with ANOVA.

### 3.5 Data collection

#### 3.5.1 Quantitative data collection

The survey data were collected online with the Webropol‐tool from 4 September to 8 November 2020. The questionnaire contained a subscale from the Calling and Vocation Questionnaire (CVQ; Dik et al., 2012) that measured perceived calling (CVQ‐Presence). CVQ‐Presence had 12 items and it measured three dimensions of calling, with four items in each: transcendent summons, purposeful work and prosocial orientation. An example of an item was ‘The most important aspect of my career is its role in helping to meet the needs of others’. The possible answers for the items were: one for not at all true of me, two for somewhat true, three for mostly true and four for absolutely true. The subscale scores ranged from 1 to 16. The total score was the mean of the three subscales. The background characteristics included the respondents' gender, age (under 35, 35–55 and over 55 years), education (vocational, Bachelor's, Master's degree or higher) and type of employment contract (permanent or temporary). We also collected information about their self‐rated health using a five‐point Likert scale: good (4–5 points) or weakened (1–3 points). Work ability was measured from 0 to 10 with the Work Ability Score as: good (8–10 points) or weakened (0–7 point). Job satisfaction was measured with one question that used a four‐point Likert scale that was divided into good (3–4 points) and not so good (1–2 points).

#### 3.5.2 Qualitative data collection

There were five phases to developing the semi‐structured interview guide (Kallio et al., 2016). Once we had decided that using semi‐structured interviews was the best method, we conducted the literature review and formulated a tentative interview guide based on it. We then pre‐tested the guide with three nurses and clarified the questions. The finished guide (Table S1) comprised questions about the nurse's career and any calling. The broad data generated by the career question will be reported elsewhere. Individual, remote interviews were conducted by one researcher (H.K. or M.H.) by video conferencing or telephone in March 2021. The recorded interviews lasted for a mean of 52 min (range 30–80) and this produced 19 h and 50 min of coverage, which was transcribed to 308.5 pages of single‐lined text, with standard Word margins in 12‐pt Calibri.

### 3.6 Data analysis

#### 3.6.1 Quantitative analysis

The quantitative data analysis used analysis of variance (ANOVA) to compare the average levels in calling between the 7780 respondents who stated their occupation. Values of *p* ≤ .05 were statistically significant. We then used ANOVA to explore the calling of the 414 registered nurses by age and education and the Student's *t*‐test to compare their gender, type of employment contract, health, work ability and job satisfaction. There were only 1–5 missing values for each of the variables of interest and these cases were excluded from the analyses in question. Frequencies and crude numbers were presented for the background characteristics of the registered nurses. We analysed the quantitative data using SPSS software, version 27.

#### 3.6.2 Qualitative analysis

Thematic analysis was used for the qualitative data (Braun & Clarke, 2006) and it was organized using NVivo software, version 12. We began by familiarizing ourselves with the data and obtaining a sense of the whole. Initial codes emerged and these were used to extract the expressions from the transcripts. By collating similar expressions together, we created sub‐themes and then main themes (Braun & Clarke, 2006). We also extracted quotes to illustrate the findings and the numbers that appear after these refer to the coded participants.

### 3.7 Rigour

Validated instruments were used to collect the survey data on calling, health and work ability. The test–retest validity for the CVQ has demonstrated good fit and construct validity (Dik et al., 2012). Self‐rated health is the most frequently used health indicator in population surveys, as it reliably predicts mortality (Lorem et al., 2020) and is a good proxy for more objective health (Garbarski, 2016). The Work Ability Score has found to be reliable (Kinnunen & Nätti, 2018) and correlate with health, burnout and leaving the profession among nurses (Ebener & Hasselhorn, 2019). We aimed to enhance the trustworthiness of the qualitative study by using a carefully developed and reported interview guide (Kallio et al., 2016) and presented original quotations to demonstrate the abstraction process. The research team worked closely together to finalize the data and followed the checklist of Good Reporting of Mixed‐method Studies (O'Cathain et al., 2008) when writing the paper.

### 3.8 Ethical considerations

We obtained research permission from all the organizations that recruited our respondents. According to Finnish legislation, this type of study does not need ethical approval (Ministry of Social Affairs and Health, 1999). We provided study information by email and verbally and this included the voluntary nature of the study and the participants' right to withdraw at any time (Finnish Advisory board on Research Integrity, 2012). We obtained written, informed consent before both data collection phases.

## 4 RESULTS

### 4.1 How the nurses responded to survey questions about calling

There were 7780 respondents (93% women) with a mean age of 48 (range 18–74) years. Their educational levels were vocational (78%), Bachelor's degree (19%) and Master's degree or higher (3%). When they were compared with the other professional groups, registered nurses had the lowest levels in all the dimensions of calling (Table 1). The transcendent summons dimension was lowest of all of the professional groups and the prosocial orientation was the highest (Table 1).

The 414 registered nurses (93% women) had a mean age of 47 (range 22–74) years and 88% had a Bachelor's degree in nursing. The majority (75%) were in permanent roles and 25% were temporary workers. Student's *t*‐tests were applied to compare the levels of calling by age, gender, type of employment contract, health, work ability and job satisfaction. Older nurses perceived significantly (*p* = .002) higher levels of calling than younger ones and those with good health (*p* = .006) and good work ability (*p* = .013) had significantly higher levels of calling than nurses with weakened health and work ability. Having a calling was also significantly higher among nurses who were satisfied with their jobs (*p* < .001) than those who were not. Gender (*p* = .191) and the length of their working contract (*p* = .880) were not significant when it came to perceiving a calling.

### 4.2 How nurses expressed their perceptions of calling in the interviews

The interview study comprised 23 nurses with a mean age of 48 (range 29–72) years. They had worked as registered nurses for a mean of 17 (range 1–50) years: 18 had received additional training in the health field (Table 2) and 12 had taken part in education in other sectors. The group included participants from both public and primary healthcare and two of them worked outside the health sector (Table 2).

**TABLE 2:** The interview participants' background information

	*n*
Other education in health field (*n* = 13)	
Primary nurse	9
Public health nurse	2
Housekeeper	2
Current workplace (*n* = 23)	
Hospital	9
Health centre, home care	7
Nursing home	3
Foundation	2
Other than health sector	2

### 4.3 Having a calling made work meaningful

Nurses said that calling made work meaningful. The nurse's personal experiences affected how motivated and engaged they were at work. Calling was *a desire to work as a nurse* and made the nurses feel that nursing was interesting work that they were suited to.

Calling is an experience of what I do does matter. (Nurse 13)

Participants said that finding work meaningful could *strengthen work engagement*. However, they also stated that nurses were more engaged in their profession a few decades ago and that today's nurses were more likely to consider nursing as one of the options they could pursue. One participant also observed that morals and quality had declined in recent years.

Before, people considered their own workplace important, even if it wasn't very satisfying … younger folk vote with their feet if something doesn't please them. (Nurse 2)

According to the participants, having a *calling could empower them to cope with work demands* and protect them from cynicism. However, calling was also seen as a risk, as it could lead to fatigue and burnout. Participants said that nurses who had a calling often had high morale and were conscientious and responsible. They stated that time and resources were scarce in hectic care environments and said that nurses who had a calling did not always act in their own best interests when they strove to meet their own high‐quality aims. They also brought up that having a calling could also risk the health of colleagues if nurses came to work when they were sick.

Calling means that I want to mitigate suffering and help others. And then when the realities don't meet the patients’ needs, you do more than you can bear, because you try to survive with scarce resources. (Nurse 12)

According to the participants, nurses with calling had a *will to learn and develop* their own professional competency and deal with work‐related issues. Two participants who said they were called to nursing had developed patient care and wider working life‐related policies and practices for nurses in their organizations.

Calling is respecting our own competency and our passion to learn new things. (Nurse 14)

According to nurses, calling also *affected the whole working community*. Nurses who found their work meaningful, and were engaged and responsible, were often considered pleasant colleagues and team players. They were also described to be the ones who created a positive atmosphere and a sense of community. However, some nurses said that nurses who had a calling could give the impression that they were superior to colleagues or that they had the right to criticize others' work. On the other hand, nurses said that their colleagues, who they considered not to have a calling, were often negative, unresponsive and even rude towards colleagues, and described situations in which these nurses only performed the bare minimum at work. This had caused resentment among colleagues and could hinder work quality.

Nurses who don't have a calling do what is necessary, not what is best in the long run. (Nurse 14)

### 4.4 Calling was humane and provided a holistic work orientation

The nurses said that having a calling resulted in a humane and holistic work orientation. It referred to nurses wanting to be present, empathic and providing holistic care for patients. Nurses said that the ones who had a calling were *present with patients*, as they were willing to help, cure and do good for others and engage with patients and show an interest in them. One participant said that nurses who did not have a calling were more likely to be interested in associating with colleagues than patients.

Have a calling is feeling that I am fully here with this person. Not half there and not thinking about something else. (Nurse 22)

Participants said that nurses with a calling showed *empathy towards patients* and were able to place themselves in other's position. This provided a sense of equality between the nurse and the patient and empowered patients. Nurses connected the lack of calling to the risk of objectifying patients and performing patient‐related tasks in a distant and mechanical way. They suggested that nursing provides wide‐ranging work opportunities and that nurses who did not have a calling might find technical duties more suitable.

Sure, you can give vaccinations or take blood samples or do some other technical work like this and it doesn't harm people. (Nurse 11)

Having a calling also manifested as a *holistic care approach*. Participants said that nurses who had a calling were often interested in advancing patient's health and lives comprehensively, guiding them and involve the patient's family in a care situation. However, there were also some participants who said that nurses could be willing to help others even if they did not have calling.

I aim to take care of the person as a whole, not just their unwell leg … if she lives with a spouse, I can ask them whether they have applied for caregiver's support. (Nurse 4)

### 4.5 Calling changed during a professional career

Nurses said that experience of calling changes during a nurse's professional career. It starts and strengthens but could then weaken and even fade away. The factors that had an impact on it were personal tendencies and character, personal values, a nurse's view of the world, their personal and work life experiences, education and training and their general appreciation of nursing.

Participants discussed the impact a nurse's *personal tendencies and character* had on whether they felt called to nursing. They considered certain inborn qualities that were needed for nursing, such as accuracy, a people‐oriented mindset and being able to handle pressure and body secretions. This meant that the nursing profession wasn't suitable for everyone. Nurses also mentioned that people had personal *values and views of the world* that they considered were related to having a calling to nursing. These were often referred to as having a range of Christian values.

I have many nurses in my team who are really suited to nursing. (Nurse 19)

The nurses also said that *personal life experiences*, such as traumas and crises in their own lives or close circles, were likely to lead to them having a calling. On the other hand, one participant said that having a good, calm life may also make people feel that they wanted to share that feeling with others. The participants also said that *working life experiences* had an influence on a person's experience of calling. Although growing competencies and successful experiences were connected to a strengthened calling, a poor working atmosphere could weaken it.

If the work atmosphere is poor, people avoid liabilities and are tired and cynical. I think that these are the kind of things that kill what I perceive to be calling. (Nurse 13)

According to the participants, also *education and training* influenced calling. They mainly referred to workplace training and how this increased their interest in new areas of nursing. However, the participants also talked about the negative influences that bad practical training experiences could have. Training instructor's negativity towards a student was considered particularly harmful for the students' perception of the profession they were about to choose. On the contrary, participants saw that if a nurse was exposed to a *general appreciation for nursing*, this could have an impact on the nurse's own ideas and respect for nursing as a profession, thus shaping their calling.

If there are a lot of positive or negative issues voiced by a certain group of professionals, it could affect how people think, like this could be a cool profession or they would never go into that kind of work. (Nurse 3)

### 4.6 Having a calling was oppressive

Interviews showed that having a calling to nursing could be oppressive, because of how it was seen today. The participants said that in the past nurses were seen as altruistic servants and that having a *calling distorted the image of nursing*. They said that the resilient image of nursing as ‘a calling profession’ has been used as a general reason for nurses' poor working conditions and salary. They had also experienced that seeing nursing as a calling reinforced nurses' subsidiary role in relation to physicians. Having a calling was not discussed in nurses' workplaces, because of the negative features that have been connected to calling.

That word calling has become kind of a taboo … people are afraid that if they talk of calling, that will have an effect on general attitudes and salary. (Nurse 21)

On the other hand, participants said that *calling reduced a nurse's self‐image* and that nurses who had a calling were often tolerant and sacrificial and not eager to stand up for their rights. They said that nurses with a calling could this way prevent the development of nurses' working conditions in general. The participants also said that nursing education had often shaped these kinds of modest attitudes.

You tolerate faults longer when you have a calling. (Nurse 17)

## 5 DISCUSSION

The results of this mixed‐method study provide important information on how registered nurses perceived having a calling in contemporary nursing. The quantitative part of the study indicated that calling was lower in nursing than in other groups of care professionals and that younger nurses had a lower level of calling than older colleagues. In general, nurses adopted a negative stance against having a calling. The qualitative investigation examined the reasons for this negativity and showed that nurses linked calling to nurses' oppressive role and poor working conditions and salary. On the other hand, interviewed nurses perceived calling as beneficial for individuals and the wider community in many ways. Our statistical findings supported this, as they showed that nurses who had a calling reported better health and work abilities and higher work satisfaction.

Having a calling in nursing has often been connected to altruistic motives of placing the needs of others first (Carter, 2014), but our findings also emphasized the benefits for the nurses themselves. Calling undoubtedly contributes to a nurse's job satisfaction because it makes their work meaningful. We found that having a calling influenced how the nurses in our study coped, and engaged, with their work and their relationships with colleagues. It also provided wider benefits to their working community, organization and society, due to their engagement and high quality of care. Unlike Carter's study (2014), our findings showed that having a calling to nursing was often part of how a nurse developed their work. For example, nurses who had a calling seemed to be more interested in developing how patient care issues were handled, as they were of significant interest to them. Not least, patients benefitted when nurses with calling adopted a humane and holistic approach (Carter, 2014; Emerson, 2017; Prater & McEwen, 2006). Thus, our findings strengthen the previous perceptions of prosocial orientation as a central element of calling (Dik & Duffy, 2009).

However, our findings also revealed the reverse side of nurses' experiences towards calling, as they connected it to nurses' subsidiary role, poor working conditions and low pay. They saw calling as a historical concept and experienced that as people still perceive nursing as a ‘calling profession’, it has led to exploitation of nurses. Recent studies have shown that nurses have described disrespect from their organizations and society, such as belittling comments, commands (Laminchhane & Bae, 2020), empty promises (Donahue, 2020) and comments on social media. Low salaries have been seen as discrimination and nurses have stated that one reason for this has been that people assume that nurses enter the profession because they have a calling for it (England, 2005; van der Cingel & Brouwer, 2021).

On the other hand, our participants perceived that nurses who had a calling were more likely to accept adverse working conditions or keep quiet about them. These finding were similar to previous studies that reported that nurses who had a calling were less likely to show negative emotions (Afsar et al., 2018). Our study found that nurses with a calling accepted situations that others would normally complain about, but then went on to develop adverse work‐related issues. This was an obvious area of conflict. Meleis (1997) stated that the traditional image of nursing as an altruistic women's task had such a strong impact on nurses' professional identities that it prevented them from thinking critically. Meanwhile, Carter (2014) stated that nurses who had a calling displayed ‘a passive docility or strict obedience’, which questioned their freedom and self‐determination. The increased attention paid to nurses' working conditions (McDermid et al., 2020) and rights (Kangasniemi et al., 2014) has increased nurses' awareness of them and could have made it easier for them to speak out. The negative consequences that nurses linked to calling could explain why the nurses in our study were less likely to have a calling than the other groups of professionals who took part. It may also be why the younger nurses had stronger negative attitudes about having a calling.

We found that a nurse's calling could change throughout their career. This highlights the opportunity that they could actually discover their calling and find nursing meaningful along the work. Remuneration issues (also Fité‐Serra et al., 2019; Halcomb et al., 2018), workload and other work environmental factors (McDermid et al., 2020; McHugh & Ma, 2014) have been recognized as central to a nurse's working life. These have all been shown to have an impact on the wider experience of meaningful work, which is pivotal for people's psychological and work well‐being (Martela & Pessi, 2018). Our findings also emphasize the fact that some nurses enjoyed interacting with patients more than others. This highlights the need to guide nursing students during their degree education, so that they find the specialty that is most suitable for their personal qualities.

### 5.1 Limitations

The limitations of this study were linked to the survey response rate, the analysis of the interview data and the data collection sequence of this mixed‐method design. The first limitation was the 9% response rate to the survey, but this was not a difficulty confined to this study. Low response rates to untargeted surveys that use the total sampling method have recently become problematic. That is why we analysed the age, gender and educational levels of the first 100 and last 100 people who responded to the whole survey, as the last respondents were more likely to resemble those who did not participate (Jooste et al., 1990). However, we did not find any differences and this indicates a probable lack of non‐response bias. The characteristics of all the respondents to the initial survey were in line with Finnish care professionals in general. Second, the interview participants tended to have strongly opposing perceptions of calling, as some favoured it and some were against it. This made it challenging for the analysis to present a coherent overall picture of the participants' perceptions. However, we believe that we have reported these diverse results accurately and transparently. Third, the mixed‐method design meant that we carried out the survey before the interviews. During the interviews, several nurses said that delving into the topic and discussing it had made them more aware that having a calling could also have positive effects on the working lives of nurses in general. This means that the findings could have been different if we had carried out the interviews before the survey. However, this would not have been practical, as the survey helped us to identify suitable interviewees.

## 6 CONCLUSION

This study indicated that a lower percentage of registered nurses had a calling compared with other care professionals. It also highlighted nurses' perspectives on their calling to the profession and the problems that they experienced in their working lives which they linked to calling. The findings have implications for how society sees the development of the health sector in general and nursing in particular. We found that calling had multidimensional benefits for individuals, patients, working communities, organizations and the wider society. However, our participants experienced that the image of nursing as a calling distorted the conception of nursing as a profession and, thereupon could have a negative impact on their working conditions. It is crucial to promote a more accurate image of nursing and improve nurses' working lives, so that nursing is more meaningful and attractive. To our knowledge, this was the first mixed‐method study to be carried out on this subject and it produced a comprehensive insight into nurses' perceptions of calling. This can help to inform further research on how we can ensure that nursing is experienced and seen as a meaningful career.

## CONFLICT OF INTEREST

The authors have no conflicts of interest to declare.

## AUTHOR CONTRIBUTIONS

All authors have agreed on the final version and meet at least one of the following criteria (recommended by the ICMJE*): (1) substantial contributions to conception and design, acquisition of data or analysis and interpretation of data; (2) drafting the article or revising it critically for important intellectual content. *http://www.icmje.org/recommendations/.

### PEER REVIEW

The peer review history for this article is available at https://publons.com/publon/10.1111/jan.15157.

## Supporting information


Table S1

## Data Availability

We do not wish to share the data.
