# Supplementary material for: Registered nurses' perceptions of having a calling to nursing: A mixed‐method study
Source: J Adv Nurs. 2022 Feb 21;78(5):1473–82. doi: 10.1111/jan.15157 (PMC9306482; doi:10.1111/jan.15157)
Supplement: Supplementary file 1 — Table S1 [file JAN-78-1473-s001.docx]

Supplementary file 1. The semi-structured interview guide

| **Opening the discussion (‘warm-up question’)** |
| --- |
| - What was it about the subject of the study that made you want to participate in this study? |
| **Theme 1. Nurse’s career choices and plans** |
| - What kind of issues made you to choose the nursing profession? |
| - What made you to choose your current workplace? |
| - What kind of thoughts and plans do you have in relation to |
| - your career |
| - studying |
| - the end of your career / retirement |
| - What do you think about your nursing career? What is it like? |
| - How is your nurse’s career influenced by |
| - employment relationships |
| - your workplace |
| - your competency and the development of your competency |
| - other issues |
| **Theme 2. Calling in the nursing profession** |
| - What do you think about calling in the nursing profession? What is it? |
| - How does a professional calling arise? Do nurse need a calling? |
| - How can you tell whether a nurse has a calling or not? |
| - in their daily work |
| - with patients |
| - in employment, such as in relationships with colleagues and ancillary workers and in teamwork |
| - in relation to wellbeing at work |
| **Theme 3. Own calling** |
| - What kind of role has calling played in *your* career choices? |
| - Has the experience of calling somehow changed during your career? How? |
| **Closing discussion** |
| - Is there something else you would like to add? |
